# A novel hydro-pneumatic fluid percussion device for inducing traumatic brain injury: assessment of sensory, motor, cognitive, molecular, and morphological outcomes in rodents

**DOI:** 10.3389/fnmol.2023.1208954

**Published:** 2024-01-16

**Authors:** Alberto Morales-Villagrán, Juan C. Salazar-Sánchez, Gustavo A. Chiprés-Tinajero, Laura Medina-Ceja, Jorge Ortega-Ibarra

**Affiliations:** ^1^MexBio Research Innovations S.A. de C.V., El Salto, México; ^2^Laboratory of Neurophysiology, Department of Cellular and Molecular Biology, CUCBA, University of Guadalajara, Zapopan, Mexico

**Keywords:** fluid percussion electronic control, H&E stain, molecular biomarkers, Morris water maze, novel FPI device, traumatic brain injury

## Abstract

**Introduction:**

The fluid percussion method is widely used to induce brain injury in rodents. However, this approach has several limitations, including variability in the resulting damage, which is attributed to factors such as manual control of the mass used to generate the desired pressure. To address these issues, several modifications to the original method have been proposed.

**Methods:**

In this study, we present a novel device called the Hydro-pneumatic Fluid Percussion Device, which delivers fluid directly to a lateral region of the brain to induce injury. To validate this model, three groups of male and female rats were subjected to lateral fluid percussion using our device, and the resulting damage was evaluated using sensory, motor, and cognitive tests, measurements of serum injury biomarkers, and morphological analysis via cresyl violet staining.

**Results:**

Our results demonstrate that this new approach induced significant alterations in all parameters evaluated.

**Discussion:**

This novel device for inducing TBI may be a valuable alternative for modeling brain injury and studying its consequences.

## Introduction

Brain trauma can be caused by diverse events, including accidents or violent acts, which can result in death or disabilities that last a lifetime. Approximately 69 million individuals are estimated to sustain a traumatic brain injury (TBI) each year (Dewan et al., 2019). The medical and economic consequences have a significant cost for any society across the world. To study the effects, mechanisms, and possible therapies of TBI, several models have been developed, including the lateral fluid percussion (LFP)-induced brain injury (LFP), lateral controlled cortical impact injury (CCI) with their pneumatic variants (Lighthall, 1988) and electromagnetic variants (Brody et al., 2007; Onyszchuk et al., 2007), and the weight drop injury (WDI) (Feeney et al., 1981), among others. The FPI model is the most established and commonly used, although it could be improved for a better understanding of the consequences of TBI in humans. The development of any other model cannot be excluded, particularly if such a model improves the efficiency in the control of the main parameters for producing TBI, for example, the peak pressure and its duration which serve to control the injury severity, not to mention the feasibility of being implemented without the need for intensive training, among other improvements.

Complete control of the severity of brain injury would be the best feature of an ideal TBI model, so any additional approach that improves the features of existing models would help to better understand the basic mechanisms as well as to design the best therapeutic strategies. Although LFP model is the most widely used and well-characterized of non-penetrating and non-ischemic TBI (Katz and Molina, 2018), there are some problems in this model that have not been solved, including the inherent characteristics of the piston that needs to be lubricated frequently, regardless of the material it is constructed with, because friction affects the pressure in the plexiglass cylinder containing the saline solution. In addition, the flexible tubing that is generally used can absorb some of the pressure, and the mechanism of releasing the mass to hit the piston requires skills from each user. In this regard, Kabadi et al. (2010) aimed to enhance the original method by introducing an air-driven impactor that employed a double-action piston pneumatics system for precise control over the impact force delivered to the embolus, thereby achieving the desired level of damage intensity. While the release of the impactor was electronically regulated, the underlying principle remained akin to inducing a pressure wave in the form of a fluid bolus. Meanwhile, Ouyang et al. (2018) made modifications to the original design to address the challenges associated with the pendulum model and aimed to eliminate the necessity for manual manipulation of this device. These authors replaced the plexiglass tube with a stainless steel cylinder and incorporated the use of an electromagnetically controlled protractor to precisely align the pendulum and subsequently strike the embolus, achieving the desired pressure to induce brain damage. On the other hand, the controlled cortical impact (CCI) model provides an alternative approach to induce varying degrees of damage by utilizing an electromagnetic piston to directly impact the dura (Brody et al., 2007; Osier and Dixon, 2016). This model allows for electronic control over parameters such as velocity, acceleration, angle, and impactor penetration. Consequently, it generates a more focal form of damage, leading to distinct morphological and behavioral outcomes that may differ from those produced by the LFP models. Therefore, our study is primarily aimed at comparing the advantages of this innovative TBI device with other fluid percussion devices.

In addition, TBI has been classified by the Glasgow Coma Scale as severe, moderate, and mild, as well as by the result from computer tomography as positive and negative abnormalities (Capizzi et al., 2019). It is well known that after the initial damage in TBI models (hemorrhage, meningeal injury, necrosis, among others), different biochemical and molecular alterations appear, such as excessive glutamate release, overstimulation of NMDA glutamate receptors, an increase of reactive oxygen and nitrogen species, and increased proinflammatory molecules. Recently, studies have focused on determining alterations in several molecules that could reflect tissue damage in both neurons and glia, and they can even appear in the blood due to the temporary disruption of the blood-brain barrier. Some of these molecules have been identified as possible biomarkers, which could coincide with the severity of TBI, and they can be present even with a non-obvious altered neuroimage. Vos et al. (2010) have demonstrated considerable increases in GFAP and S100B after TBI, and thus these proteins could be considered strong biomarkers. In addition to these proteins, neurofilaments have been found elevated in three experimental models of TBI (Yang et al., 2019). Several proteins derived from both glia and neurons have been evaluated as putative biomarkers, however, only GFAP and ubiquitin c-terminal hydrolase L1 (UCH-L1) are cleared by the FDA (2018) to aid in the assessment of middle-acute TBI. It is interesting to have for the first time this approval and no doubt additional proteins will be added although the method and devices used for this purpose need to be validated first in different experimental models of TBI.

Therefore, in this work we introduced a novel device to induce TBI with the principle of producing a pressure wave over the dura with a fluid bolus release it electronically from a stainless container using solenoid valves controlling the speed and the intensity of the pressure wave, the model validation was assessed by studying the morphological, behavioral alterations and the expression of some putative biomarkers of brain damage.

## Materials and methods

### Device design and construction

**FIGURE 1:**
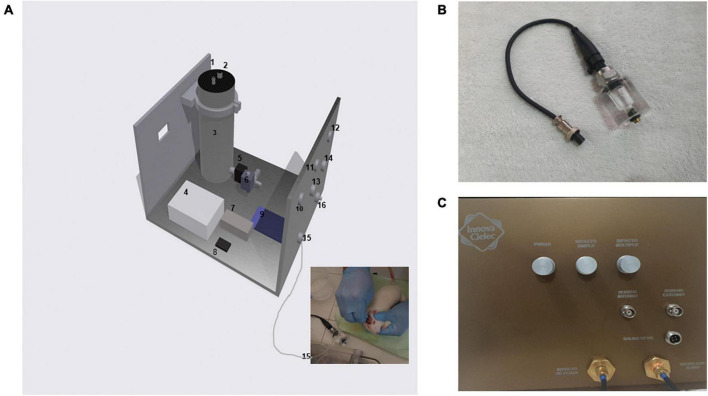
New TBI device. **(A)** Components of the hydropneumatic device: (1) Sealed input liquid, (2) Air path inlet for setting desired pressure, (3) Stainless steel container, (4) 12-volt DC power supply, (5) and (6) Liquid solenoid valves, (7) Solid-state relay, (8) 5-volt DC power supply, (9) Microcontroller, (10), (11), and (12) Push buttons for purge, single and multiple hittings, (13) and (14) Analog outputs for pressure transducers, (15) Outlet liquid for hitting, (16) Liquid purge outlet. The inset image corresponds to a real hitting procedure in an experimental animal. **(B)** The external pressure sensor attached to the acrylic chamber to emulate the skull cavity before attaching the Luer lock to the rat brain. **(C)** The final device with its control panel for producing hits. Please note that the tubing for inducing the hitting was only changed from the acrylic chamber (once the pressure was tested) to the rat brain to produce the TBI.

This device was designed and built with the following parts: an air compressor, a stainless steel cylinder, power supplies (5 and 12 Volts DC), two solenoid valves (normally closed, activated with 12 V DC), a solid-state relay, three push buttons, and an Arduino microcontroller (Figure 1A).

The main idea of this new setup is to maintain the impact of the fluid on the rodent’s brain with significant modifications to its principle. The use of a plexiglass container and pendulum was avoided, and instead of these two components, an airtight stainless steel cylinder was machined with the following dimensions: 80 mm inner diameter and 200 mm height with approximately 1.0-liter volume. Near the bottom, a perforation was made to place the first liquid solenoid valve, which is electronically controlled to open and close, with the purpose of releasing the fluid at a precise time (in milliseconds). At the outlet of the solenoid valve, a bronze manifold connector was attached with four ways. One end was used to place a pressure transducer perpendicularly to the direction of fluid (Figure 1A). Another outlet was used to induce the impact of fluid on the rodent or to test the final pressure with an acrylic chamber that has a second transducer attached to it. At the remaining terminal, a second solenoid valve was attached perpendicularly to the direction of the fluid to release the inner pressure every time a hitting event was applied, either to the rat or the acrylic chamber (Figure 1B). The final device with its control panel for producing hits (Figure 1C).

The sequence to produce the fluid hit was programmed as follows: the pressure was generated with an air compressor, and the final pressure was set to the desired value with a manual regulator monitored by an analog manometer. The opening and closing cycles were controlled with a microprocessor and a solid-state relay to open and close the solenoid valves. Three push buttons were used. The first was used to purge all the paths, setting an initial pressure of 0.34 atm (five PSI), leaving the two valves open for one second. This process can be repeated to eliminate the bubbles inside the system. Normally, two times were enough. The second button was programmed to open (ten milliseconds) and close (ten milliseconds) the first solenoid valve. Immediately after, the second solenoid valve received an opening signal that lasted ten milliseconds to release the remaining inner pressure. The complete cycle lasted approximately 50 milliseconds. The third button was programmed in the same way, but it allowed for multiple fluid hits (five in this test set) with a delay of one second between them. To monitor the inner pressure (right before the fluid outlet, in a similar position to that used in the pendulum model), an analog transducer was used (Walfront, model: LEPAZA60120, range 0–100 PSI, 0.5–4.5 V). The voltage signal was digitized using an Arduino platform through a short program that converts the voltage into atm values. The data displayed were captured on a PC with the Arduino library: Ardu Spreadsheet by Indrek Luuk.^1^

To ensure the pressure applied to the rodent of the desired intensity, a cylindrical acrylic chamber with a volume of two milliliters was constructed. This chamber had a pressure transducer (external transducer) at one end and a female Luer fitting at the other. Saline solution was used to fill the chamber, and care was taken to avoid the presence of bubbles. A male Luer fitting from the device, similar to the one used for the rodent, and then it was connected to induce the fluid hitting. The pressure was tested and measured simultaneously with that applied to the cylinder, several times, until both transducers showed a homogeneous value. At this point, the equipment was ready to be used in the experimental procedure. In order to compare the characteristics of the generated pressure by the hydropneumatic device a classical pendulum was also tested with the same acrylic chamber. The generated pressure was set leveling the pendulum until reaching approximately a similar pressure produced by the hydropneumatic device, several replicates were determined to evaluate the reproducibility of both devices, 15 replicates were assayed for the hydropneumatic device and ten for the pendulum.

### Animals and experimental groups

Male Wistar rats weighing between 290–400 g were used in the experimental procedure and were housed at 25°C with a 12-h light-dark cycle and given *ad libitum* access to food and water. All procedures were performed according to the Mexican Research Norms NOM-062-ZOO-1999 and approved by the local animal care committee of CUCBA, University of Guadalajara, to minimize the number of animals used and their suffering. Prior to the experiments, the animals were acclimated for one week. Sensory and motor studies were performed before the surgical procedure. The animals were randomly allocated to different experimental groups: intact control group, sham group, and experimental group (applying 2.0 atmospheres of pressure using the LFP device).

### Surgical procedure and TBI induction

The animals were first anesthetized with 4 percent isoflurane inside an acrylic chamber. Then, they were placed in a CNC motorized stereotaxic frame (INNOVA-CIELEC, S.A. DE C.V. Tonala, Jalisco, Mexico) and the isoflurane concentration was set at 2.0 percent throughout the surgical procedure. A circular hole was made over the skull with a 4.0 mm diameter trephine at (AP 4.0, ML −4.0 from Bregma). The trephine was mounted on a motor drill and joined to the Z-axis of the stereotaxic frame. The trephine was gradually lowered in several steps (Z-axis), with the first step of 500 microns and three or more additional steps of 100 microns depending on the thickness of the skull in each animal, followed by steps of 10 microns to reach the dura without disrupting it. The final removal of the trepan was done manually using a stylet to remove any remaining bone and leave a circular hole, while maintaining the integrity of the dura. The surface of the skull was dried and scratched with a stylet to allow for better adhesion of a female Luer fitting that was placed over the hole and fixed in place with three stainless steel screws. Acrylic cement was then cast over the screws to ensure a stronger adherence of the Luer fitting to the skull, thus preventing any leaking. After the surgery, the animals were allowed to recover for 90 minutes before being anesthetized again to induce TBI. The outlet tubing from the device was connected and the hitting fluid was applied by pushing the yarnell programmed button (2.0 atmospheres, 50 milliseconds).

### Sensory and motor assays

To assess the effects of TBI induction, sensory-motor studies were conducted using a neuro-score scale adapted from Yarnell et al. (2016) with minor modifications. The scale comprised ten tests that evaluated both motor (1–5) and sensory (6–10) abilities. Each test was scored with values of 0, 1 or 2, based on the following criteria: 0 for optimal response, 1 for poor response, and 2 for no response. The type of response considered for each category depended on the characteristics of the test, as follows: (1) general balance (ability to perform on a beam), (2) landing test (rat’s response to a drop), (3) tail raise test (hind paw response to tail lifting), (4) drag test (front grasp response), (5) righting reflex (ability to right oneself), (6) ear reflex (motor ear response to an upset stimulus in ear), (7) eye reflex (blink response to an annoying eye stimulus), (8) sound reflex (movement response to a sudden noise), (9) tail reflex (tail response to pinching), and (10) paw flexion reflex (paw response to pinching hindpaw). Each test was performed three times with a ten seconds latency between each trial, and the rats were allowed 20 seconds of rest between the tests. This evaluation was carried out for five days before the rats underwent surgery (both sham and TBI groups). For the TBI group, the rats were allowed a 90-minutes recovery period under observation after surgery before the TBI was induced. Sensory-motor tests were conducted at 5 hours, 24 hours, 7 days, 14 days, and 21 days after surgery (for both sham and TBI groups).

### Morris water maze

The Morris water maze (MWM) is a frequently used test to assess learning and working memory in studies related to brain trauma in rodents (Hamm et al., 1992). The water maze consisted of a black circular pool with a diameter of 1.4 m, filled with water to a depth of 30 cm. The water temperature was maintained between 23°C and 24°C. The pool was located inside a 4.5 × 4.5 m room with dark walls, one of which had a black curtain to allow the experimenter to observe the rat while swimming. A Canon HD CMOS VIXIA HF R70 video camera was mounted on the ceiling to observe the entire surface of the pool. The pool was divided into four quadrants (NE, SW, NW, and SE), and a black painted cylindrical platform with a diameter of 10 cm and height of 20 cm made of cement and polished surface was placed 1 cm below the surface in the NE quadrant.

A visual cue was also used consisting of a 16 cm diameter yellow circle containing a red pentagonal star drawn on a white letter size sheet, which was placed on the north wall at a height of 1.40 m. The visual cue was attached to the wall so that it was directly in front of the submerged platform.

During the test, the rat was placed in the quadrant opposite to the one containing the platform with its head pointing toward the wall. The experimenter then exited through the curtain and waited until a second experimenter, who observed the pool through a hole in the curtain, indicated the moment when the rat found the platform and remained on it for ten seconds. The first experimenter then removed the rat from the water, dried it with a towel, and returned it to its respective box. If the rat did not reach the platform within two minutes, the second experimenter asked the first experimenter to help the rat find the platform. Once the rat remained on the platform for ten seconds, the experimenter removed it from the water, dried it with a towel, and returned it to its respective box.

The test was repeated five times with a resting time of three minutes between each trial to complete one session. One session was performed per day for three consecutive days as a habituation period. The fourth day was used for rest, and the fifth day was designated as the evocation day, performed under the same conditions as days 1, 2, and 3. The sixth day was the test day, which involved removing the platform from the pool and leaving the rat in the pool for one minute under the same conditions as the previous days. All sessions were video-recorded, and during the habituation period (days 1, 2, and 3), the total time to find the platform was the parameter evaluated. On the evocation day, spatial memory was evaluated through three tests: the total time to reach the platform, the time that the rat remained in the opposite quadrant, and the time remaining in the correct quadrant (which contained the platform). On the test day, the relearning parameter was determined, and the time registered was that of the rat remaining in the right and wrong quadrants. The MWM was performed at 5 hours, 24 hours, 7, 14, and 21 days after surgery (sham group) and TBI (TBI group).

### Nano dot blot procedure

The Nano dot blot procedure was performed to investigate the impact of TBI on injury biomarkers in peripheral blood. Temporal and consecutive samples were collected from the tail before (pre-surgery) and after TBI induction (at 3, 9, 14, and 21 days). The blood was centrifuged at 3000 g for 15 minutes, and the serum was separated and stored at −30°C until use. The nanodot blot analysis was carried out following the protocol described by Ortega Ibarra et al. (2021). In brief, the serum samples were diluted 1:50, and five syringes were loaded with the samples to spot different patterns of dots on nitrocellulose paper. The dots were immediately blocked with an albumin solution (2.5%). Subsequently, to detect the presence of various injury biomarkers, washing and 18 hours of incubation with primary antibodies and appropriate secondary antibodies were performed, and the chemiluminescence produced was quantified (Table 1).

**TABLE 1:** Antibodies used in the nanodot blot technique to measure injury biomarkers.

Primary antibody	Description	Amounts/dilution	Supplier
**Anti-NF-L**	Neuro filament light found in damage axons of neurons	250 μL (dilution 1:2500)	(Abcam,ab223343)
**Anti-GFAP**	Glial fibrillary acidic protein, synthesized by astrocytes in an astrogliosis process	250 μL (dilution 1:2500)	(Abcam, ab68428)
**Anti-PSD95**	Protein member of the membrane, regulates the trafficking and localization of the glutamate receptors	250 μL (dilution 1:2500)	(Abcam, 76115)
**Anti-S100B**	S100 calcium binding protein B is astrocytes- specific and is usually elevated due to nervous system damage	250 μL (dilution 1:2500)	(Abcam, 41548)
**Secundary antibody**	**Description**	**Dilution**	**Supplier**
**Biotinylated secondary antibody made in goat**	To bind to primary antibody to produce revealed reaction	Dilution 1: 50	(Abcam, ab64264)

### Hematoxylin and eosin staining

At 90 days after TBI induction, the animals were anesthetized by intraperitoneal injection of Nembutal (60 mg/kg, i.p.) and transcardially perfused with phosphate-buffered saline (PBS, 0.1 M, pH 7.4, 37°C), followed by 4% paraformaldehyde (in 0.1 M PBS, pH 7.4). Then, the animals’ brains were removed and post-fixed for 72 hours at 4°C to prevent possible autolysis. The brains were then mounted on a vibratome (WPI, FL, USA) and sliced coronally at a thickness of 30 μm. These sections were collected consecutively in separate wells of an incubation chamber containing PBS. Next, the brain slices were mounted on gelatinized slides and allowed to dry at room temperature. After that, the brain slices were hydrated with three changes of 100% ethanol for two minutes per change and transferred to 95% ethanol for two minutes and 70% ethanol for two minutes. Then, the brain slices were rinsed in running tap water at room temperature for at least two minutes. The slices were placed in hematoxylin solution for one minute and rinsed under running tap water and distilled water at room temperature for at least ten minutes per change. Then, the slices were stained in eosin solution for one minute. After staining, the brain slices were dehydrated in two changes of 95% ethanol for three minutes per change and transferred to 100% ethanol for three minutes. Finally, the brain slices were cleared in three changes of xylene for 1 minute per change and a drop of Permount was added over the brain slice on each slide followed by a coverslip. These slides were observed using a microscope to describe the damage induced by TBI, and photographs of the lesion were taken in the neocortex ipsilateral. The same procedure was repeated to the sham group.

### Statistical analysis

The normal distribution of nanodot blot and sensory-motor/cognitive data was verified with the Shapiro-Wilk test. The data are presented as mean ± SD. An ANOVA multiple comparison and Sidak *post-hoc* test were applied in case of normal distribution, while the Kruskal-Wallis test was used when the distribution of data was not normal. A significant value was considered as *p* < 0.05. GraphPad Prism 9 software was used to perform statistical analysis and create graphics.

## Results

### Hydro-pneumatic fluid percussion device

**FIGURE 2:**
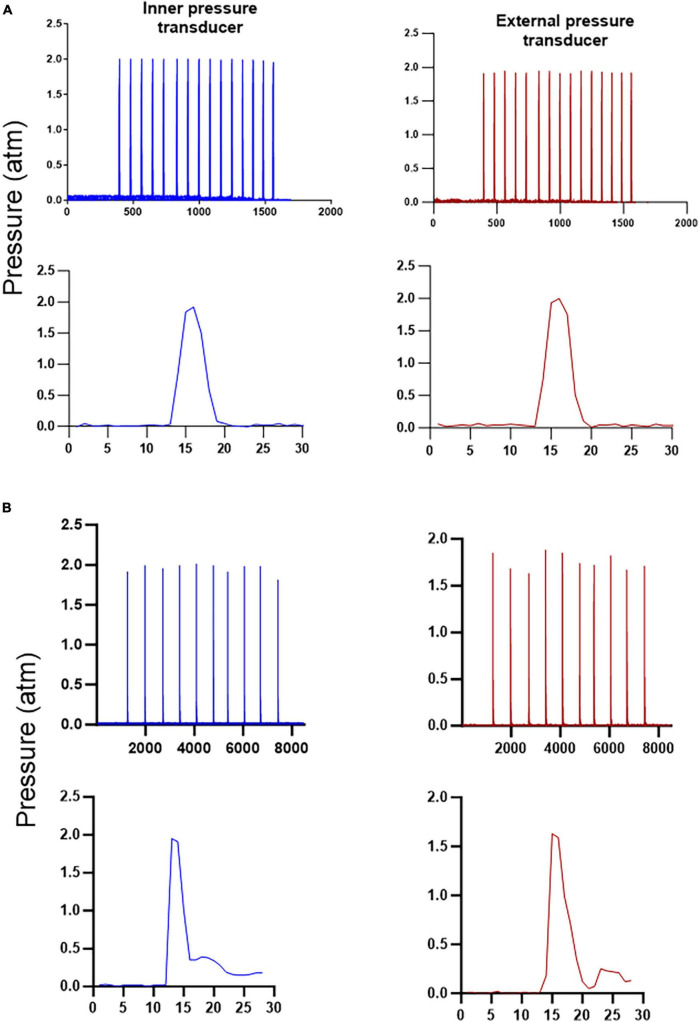
Reproducibility of the new device and classical pendulum. Graphs in **(A)** show the profile of fifteen replicates of the pressure generated electronically by opening and closing the solenoid valves and an amplification of a single test, both at the internal and external pressure transducers, with the hit lasting approximately 50 milliseconds in the case of hydropneumatic device, as can be seen the replicates show less variability than in the pendulum testings **(B)**, although the velocity of the pulse generated by the pendulum lasted only 25 milliseconds and the profile of the single pulse appear different from the hydropneumatic device.

The hydropneumatic device offers the advantage of easily purging the system of bubbles by simply pressing the purge button twice. This step opens the two valves simultaneously for one second. Subsequently, to ensure the uniformity of the pressure pulses in terms of intensity and duration, several pressure pulses were generated and monitored. In this test, the third button was pressed to obtain a pressure profile monitored by the two pressure transducers for several pulses. The initial pressure was set at 2 atmospheres, and an analog manometer was used for monitoring. The pressure recorded in the internal and external transducers averaged 1.98 and 1.92, respectively, from 15 pulses, with a variability percentage of 0.9 and 0.6, respectively. These results indicate that there was no significant loss of initial pressure. The profiles of the two sensors exhibited similar characteristics when observed in detail (Figure 2A). These findings enabled the system to be used in experimental animals. In the similar test used to compare the reproducibility of the classical pendulum under the same circumstances (Figure 2B) the average pressure generated was 2.0 and 1.81 atmospheres for the internal and external transducers and the variability was 2.9 and 5.2 percent respectively, these results show a clear difference in generating a similar pulse manually versus electronically. Another difference easily to note is the characteristics of the pulse, in the case of pendulum, it shows a small bump after a rapid increase in the pressure, this waveform is similar to that published by Ouyang et al. (2018).

### Animal surgery and weight

After trepanation, all rats retained their meninges. Immediately after the TBI procedure, all animals experienced a single tonic convulsion characterized by hind leg extension, tail extension, and stiffening, along with apnea lasting for at least five seconds. It was necessary to provide manual assistance for breathing until the rats could breathe on their own. The luer lock was then removed, and the head was sutured. The rats were allowed to recover before proceeding with the rest of the experiments as described in the Materials and Methods section. The average weight of the rats after surgery remained between 290 and 408 grams. It is worth noting that the average weight of the intact group of males remained higher than that of the other groups on almost all monitoring days.

### Sensory and motor alterations

#### Sensory alterations observed in male animals of different groups

**FIGURE 3:**
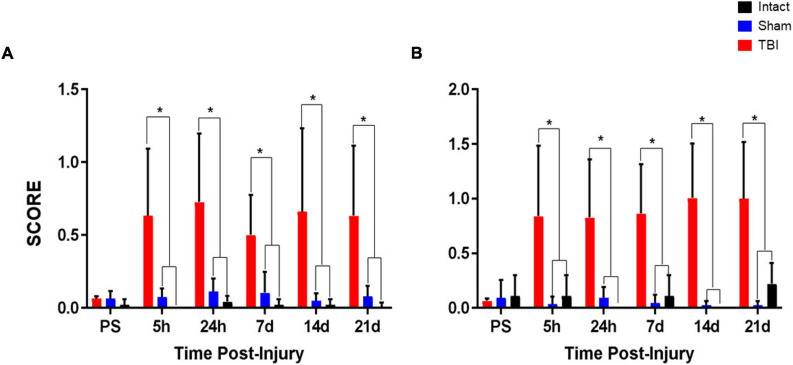
Sensory and motor tests for rats. The neurological scoring included ten tests evaluating sensory and motor abilities. Three replicates of each test were performed with a 10–20 second interval between each test. Animals were evaluated for five days prior to surgery (pre-surgery, PS). The sensory-motor tests were performed at 5 hours, 24 hours, 7 days, 14 days, and 21 days after surgery. **(A)** Graphs show the scores obtained in the sensory tests for intact, sham, and TBI groups. The TBI group had a significantly higher score than the intact and sham groups. **(B)** Graphs show the scores obtained in the motor tests for intact, sham, and TBI groups. Similarly, the TBI group had a significantly higher score compared to the intact and sham groups. *p < 0.05.

Data on the sensory performance of the rats were obtained using the methodology previously described. It is worth noting that the highest values correspond to the most unfavorable performances, and that optimally, the rats obtain scores close to zero. On the day before the intervention (surgery and injury for the TBI group, only surgery for the sham group, no intervention for the intact group), male animals of the different groups showed similar values (TBI 0.066 ± 0.013, *n* = 10; sham 0.066 ± 0.05, *n* = 7; intact 0.022 ± 0.038, *n* = 3), with no significant difference between any of the groups (Figure 3A). The same testing was repeated after the surgery or TBI at 5 hours and 24 hours, 7, 14, and 21 days. During this period, on each occasion, the TBI group of animals showed a significant deficit (*p* < 0.05) in their performance compared to the sham and intact groups (Figure 3A). Additionally, it can be observed that at 24 hours post-surgery, the highest performance deficit was reached in the TBI group compared to the other groups (TBI 0.73 ± 0.47, *n* = 10; sham 0.1137 ± 0.09, *n* = 7; intact 0.044 ± 0.038, *n* = 3).

#### Motor alterations observed in male animals of different groups

On the day prior to the surgery, all groups obtained similar values close to zero (TBI 0.066 ± 0.020, *n* = 10, sham 0.094 ± 0.161, *n* = 7, intact 0.11 ± 0.19, *n* = 3). It is observed that there are no significant differences between groups (Figure 3B). The tests were repeated after the surgery at 5 hours and 24 hours, 7, 14, and 21 days. The results corresponding to all these days showed an increase in the values obtained by the TBI group, which in all cases were significantly higher (*p* < 0.05) in relation to the sham and intact groups. It can be noted that the traumatic injury provoked in the TBI group influenced the efficiency of these rats to carry out the motor tests.

### Cognitive performance of animals

**FIGURE 4:**
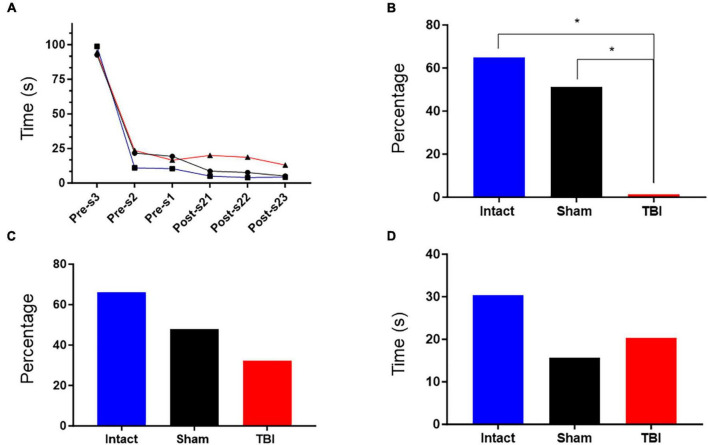
Results of the Morris Aquatic Maze (MAM). **(A)** Escape latency observed in rats from the TBI, sham, and intact groups. The escape latency was determined by averaging the time (seconds) each rat took to find the platform after being placed in the pool. Sampling points correspond to three days of training prior to surgery (pre-s at days 3, 2, and 1) and three days after surgery (post-s) at days 21, 22, and 23. *p < 0.05. **(B)** This graph shows the decrease in the percentage of time that the rat spent completing the escape task. The percentage was obtained by comparing the average total time spent completing the task during the pre-s habituation days to the average total time spent completing the task during the post-s habituation days, i.e., [100-(average time of post-s days*100/average time of pre-s days)]. **(C)** This graph shows the decrease in the percentage of time that the rat remained in the opposite quadrant. The percentage was obtained by comparing the mean time that the rat remained in the opposite quadrant on the evocation day during the pre-s phase to the mean time that the rat remained in the opposite quadrant on the evocation day during the post-s phase, i.e., [100-(mean time on post-s evocation day*100/mean time on pre-s evocation day)]. **(D)** This graph shows the time that the rats remained in the quadrant containing the platform. The value was obtained by averaging the time spent by rats in each group.

The rats were subjected to MWM according to the previously described paradigm and evaluated during habituation days 1, 2 and 3, which correspond to the three days prior to the surgery, then the habituation tests were repeated for three days, it is important to note that this second round of tests corresponds to days 21, 22 and 23 after surgery, this with the purpose of allowing the rats to a proper recovery of the wounds caused by the intervention. In this way, a tendency to decrease the escape latency time was observed in the sham and intact groups, but not in the TBI group (Figure 4A). Animals improve learning manifested in the decrease of the escape time, because of test repetition in the successive days for both sham and intact group, but not for the TBI group (Figure 4A). With respect to the percentage rates that the rats spent in completing the task, the TBI group showed a significantly lower percentage compared to the sham and intact groups (Figure 4B). In the case of the percentage rate that the rats remain in the opposite quadrant, a non-significant reduction in this percentage is observed for the TBI group compared to the rest of the groups (Figure 4C). Furthermore, when rats remain in the quadrant containing the platform, it can be observed that although the time value in seconds decreases in the TBI group, there were no significant differences with respect to the other groups (Figure 4D).

### Increases in the injury biomarkers of animals with TBI

**FIGURE 5:**
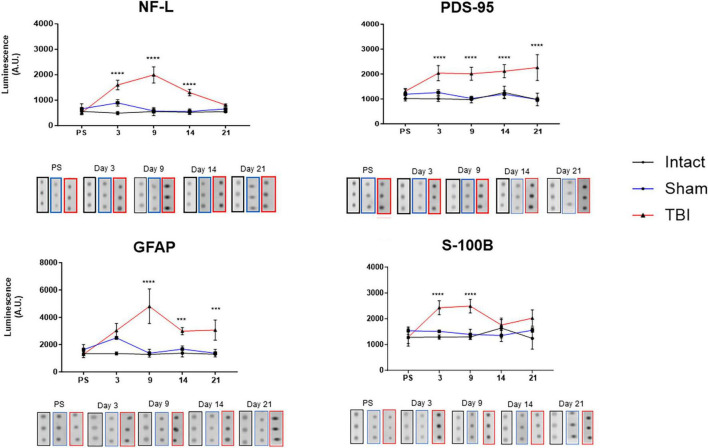
Expression of injury biomarkers. The graphs show the temporal course of injury biomarkers expression in the serum from the intact, sham, and TBI groups. The protein expression was evaluated at day one (labeled as pre-surgery, PS) and after TBI induction (3, 9, 14, and 21 days). The values are expressed as the luminescence intensity obtained from an average of the nanodots sampled over the nitrocellulose membrane, as previously described of light neurofilament (NF-L), postsynaptic density protein-95 (PDS-95), Glial fibrillary acidic protein (GFAP) and serum 100 B protein (S-100B). A representative scan of everyday tests are shown below of each graph representing the protein expression. Note a clear increase in luminescence in the TBI group with respect to the intact and sham ***p < 0.005, ****p < 0.0001.

Four putative injury biomarkers were analyzed at different time points: before and 3-, 9-, 14-, and 21-days post-surgery (Figure 5). Increases were observed in all proteins tested in serum after inducing damage with this model. However, clear differences were evident for NF-L, PDS-95, GFAP, and S-100B proteins, with the first increase detected at day three, reaching a maximum value at day nine after surgery and with a tendency to return to baseline values at day 21, when the data were compared with the intact and sham groups, with the only exception at day three for the sham group for GFAP. Similar increases for PSD 95 were determined, and their level remained constant throughout the experimental time tested, with no return to baseline levels (Figure 5). The results clearly showed that damage produced by this device is reflected in the increase in these injury markers that reached a detectable level in peripheral blood.

### Damage induced by TBI

**FIGURE 6:**
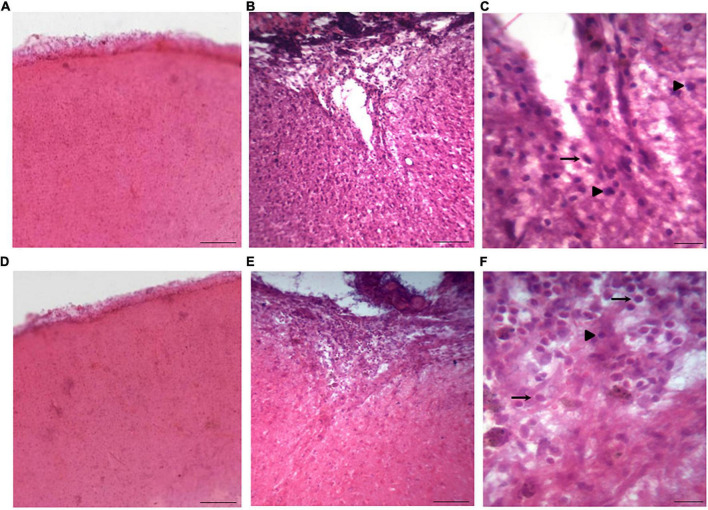
Coronal sections stained with H&E in rats from the sham and TBI groups. **(A,D)** Show representative images from the sham group with the same resolution, while **(B)** and **(E)** are pictures from the TBI group (injured rats) with the same resolution as in panels **(A)** and **(D)**. In addition, **(C)** and **(F)** are low magnification pictures from panels **(B)** and **(E)**, respectively. In the injured rats, tissue sections displayed damaged neurons around the lesion site, featuring nuclear pyknosis (indicated by head arrows) and cytoplasmic vacuolization (indicated by arrows), accompanied by tissue loss, in contrast to the sham group. The scale bar of images panels **(A,B,D,E)** corresponds to 500 μm, while scale bar of images of **(C)** and **(F)** corresponds to 100 μm.

The H&E staining was used for evaluating brain tissue loss and injury in both sham and TBI groups based on the appearance of neurons. The cells were assessed by the presence of nuclear pyknosis which is distinguishable from cell changes due to their eosinophilic appearance and blebbing of the nuclei. The TBI group exhibited brain tissue loss and damaged cells around the lesion site in injured rats compared to the sham group (Figure 6), in which mild damage to cell bodies was observed. Furthermore, the rats in the TBI group displayed nuclear pyknosis, nuclear margination, irregularity of cell structures and cytoplasmic vacuolation in cells from the cortex (Figure 6).

## Discussion

TBI is highly variable, this due to its inherent and diverse causes, for this reason there is not a perfect model that can replicate all the consequences related to a particular accident or any other trauma. Then, several models have merged to understand and to study the effects of the different forms of brain injury. LFP model has been widely used and despite of the fact that several drawbacks are attributed to this model, it is still an important reference to study the TBI, some versions of this approach have appeared trying to improve the original, this is, changing the way to induce with a better control the liquid wave inside the brain produced for the LPF (Kabadi et al., 2010; Ouyang et al., 2018). In this work, we introduced a new alternative to produce the damage over the brain in a similar way to that produced by the pendulum. The pneumatic pressure applied inside the cylinder that contains the fluid for the hitting is controlled in a better way with a single air regulator instead of leveling the classical pendulum to reach a desired pressure, which assures a more stable and reproducible way to release a force over the brain and not depending of the mass and the level in which the mass is positioned (Newton’s second law) to set the force and velocity of the hitting. The use of solenoid valves permits to release the pressure in a desired time, in this case the event lasted approximately 50 milliseconds, although is possible to program several important parameters to reproduce a desired effect, such as the intensity of the hitting decrease or increasing the pressure in a really easy way (just selecting the pressure with an air regulator), and the time that the pressure can last inside the brain. Fortunately, there are in the market more sophisticated liquid valves that have the possibility to be opened and closed in a shorter time controlled with the solid-state relay that can send a signal at one millisecond, this is not possible with the pendulum. The introduction of these changes in the original LFP model let to produce a compact device that has all the controls to produce the TBI, like the microprocessor, power supply and the analogical outlets to monitor the pressure with any data acquisition system like an oscilloscope or with a single Arduino platform used in this work, this is programing any desired pressure scale, in this case atmospheres, without the needing to convert the voltage in a further value and all the data are collected and saved in an excel data file. The variability between every testing was less than one percent, with a non-significant reduction in the pressure at the external sensor, this is where the hitting would take place tested with the acrylic chamber, as far of our acknowledge, this is the first time of the use of a chamber to test the final pressure, although the brain is not a sealed chamber, it could be a good approach to assure the final intensity of the hitting. Comparing these results with the data obtained with the classical pendulum, there is a clear difference in the profile of the waveform obtained, this was more similar between pulses with less variability and the pressure pulse generated increase and return to the baseline in a similar way, the difference with the classical pendulum could be due to the lack in the cylinder of a quick pressure release after producing the hitting, producing a delay in returning to the zero pressure.

Taking in account all these changes the hydro pneumatic device was used to study the morphological, molecular, behavioral, and cognitive effects of this new alternative of LFP model producing similar damage of that produced with other LFP devices in an easier controllable way. It is important to note that the CCI model also has the possibility to set the velocity, acceleration and force electronically, however this model does not produce the same morphological damage as the LFP model, for this reason the data obtained are not matter of comparison with this model.

In order to carry out the sensory and motor tests, it was necessary to control aspects that may elicit a stressful response in the rats, which can translate into an altered muscular response. To this end, the following considerations were taken into account for the rats: acclimatization to the cage and biotherium, constant and repetitive handling at the same time to establish a routine, sufficient food and water, and avoiding overcrowding. For the rat handlers, good training was essential to acquire skill in handling and interpreting the results in a more homogeneous way, reduce the risk of misinterpretation, and avoid holding the rat too tightly or weakly. The number of handlers was also minimized, and lotions or any substances that may disturb the rat’s sense of smell were avoided. There was a significant decrease in sensory and motor abilities of the rats subjected to TBI, which was observed over time from 5 hours to 21 days post-intervention/surgery. It should be noted that the rats in the TBI group showed considerable standard deviation, which could be attributed to several factors, including the inherent ability of each animal to assimilate a lesion. These results are consistent with previous studies using LFP, where alterations in sensory and motor tests were observed at similar times (Thompson et al., 2006; Kabadi et al., 2010; Peterson et al., 2015; Schurman et al., 2017; Ouyang et al., 2018). The presence of these alterations supports the use of the present device to induce TBI in an easy and effective way with similar sensory and motor deficits.

In the case of the results obtained in the MWM, it is important to consider that although no significant differences were found in the escape times between the different groups, there was a tendency for the TBI groups to increase the escape times once the intervention/surgery was performed. It is important to note that even a few seconds can be critical for survival. Furthermore, it should be noted that perhaps the results would have shown greater differences between groups if the Morris tests had been performed closer to the date of TBI induction. However, this was not possible as the rats needed time to heal their wounds before entering the pool. The percentage of time that the rats spent completing the task as well as the percentage of time that the rats remained in the opposite quadrant showed results that can be attributed to memory capacities, since they were performed on evocation days. In addition, no significant differences were found in all cases in the time spent in the correct quadrant. However, the principal results showed that the performance of the rats subjected to TBI was inferior to the rest of the groups, although not significantly so. These data are in line with other studies in which spatial memory was tested by MWM and showed cognitive deficits. However, it is worth noting that the difference in the parameter of time in escape latency is more noticeable in these studies (Kabadi et al., 2010; Schurman et al., 2017; Ouyang et al., 2018) than in our study. This difference may be due to the time lapse between the induction of TBI and the execution of the test and the physiological recovery processes that are set in motion after a trauma of this type, or the difficulty of clearly identifying cognitive alterations after a long period of TBI (Brooks et al., 2017); another factor that attenuated the results is the intensity of the pressure used in this work (2 atmospheres), that corresponds to a moderate damage; this is the probable reason because we did not find a clear difference between groups. However, a long study of 4 months after TBI showed significant differences between the TBI and sham group in the escape latency, which could be due to a chronic pathological process (Yasmin et al., 2022).

In addition to evaluating the effects produced by the TBI with this device on some putative injury biomarkers, such as GFAP and S-100B derived from glia, and light neurofilament and PSD95 from neurons, using the nanodot-blot technique previously reported by our group (Ortega Ibarra et al., 2021), the temporal profile of these biomarkers during the evaluated period was also studied. In general, these biomarkers had a similar temporal profile, with an evident increase in their levels since the third day after surgery, reaching a peak at day nine, and a clear tendency to return to normal values found before surgery. It has been reported that after TBI induction, a blood-brain barrier disruption occurs (Van Vliet et al., 2020), which can be damaged for several days, weeks, and even months. In this study, the biomarker expression profile could reflect a temporal disruption of the BBB that could be recovered after three or four weeks, without disproving the possibility of further damage inside the brain that may not be evident in peripheral tissue. Although the molecular biomarkers in general tend to decrease their level in serum 21 days after injury, GFAP and PSD-95 remain high in the TBI group in comparison with the sham and control groups. Despite the fact that these two biomarkers could reflect brain damage, we did not find a further increase in behavioral alterations. It is possible that additional time must be required to decrease, since a recovery process could be in progress.

In human studies on post-brain injury, the levels of several proteins have been evaluated as possible biomarkers that could reflect the lesion severity, and the remarkable role of GFAP is a clear reflection of brain damage (Cardinell et al., 2019; Czeiter et al., 2020). In addition, the results of our morphological analysis are consistent with findings from other published studies, where mild levels of damage to neuronal cell bodies were observed after TBI induced by other fluid percussion devices within similar time periods (Spain et al., 2010; Jin et al., 2017; Ouyang et al., 2018; Huang et al., 2022). Based on these results, we conclude that the present hydro-pneumatic fluid percussion device is a suitable alternative for studying injury mechanisms produced by TBI.

## Data availability statement

The raw data supporting the conclusions of this article will be made available upon request to the corresponding authors.

## Ethics statement

The animal study was approved by the local Committee for Research Projects at the University of Guadalajara. This study adhered to the official Mexican standard regulations, specifically NOM-062-ZOO-1999 and NOM-033-ZOO-1995, which delineate the “Technical specifications for the production, care, and utilization of laboratory animals” and address the proper disposal of biological waste (NOM-087-ECOL-1995). The experiments were conducted with the aim of minimizing the number of animals involved, thereby alleviating their potential suffering. The study was conducted in accordance with the local legislation and institutional requirements.

## Author contributions

AM-V designed and built the TBI device, performed TBI in animals, and wrote and revised the final version of the manuscript. GC-T performed surgeries, MWM and its analysis. JS-S performed sensory/motor tests and their analysis, and wrote the manuscript. LM-C assisted with surgeries, performed H&E staining and its analysis, and wrote and revised the final version of the manuscript. JO-I performed the nanodot blot technique and its analysis. All authors contributed to the article and approved the submitted version.

## Footnotes

1
https://circuitjournal.com/arduino-serial-to-spreadsheet
